# Supplementary material for: Ultra-thin solid electrolyte interphase evolution and wrinkling processes in molybdenum disulfide-based lithium-ion batteries
Source: Nat Commun. 2019 Jul 22;10:3265. doi: 10.1038/s41467-019-11197-7 (PMC6646323; doi:10.1038/s41467-019-11197-7)
Supplement: Supplementary file 1 — Supplementary Information [file 41467_2019_11197_MOESM1_ESM.pdf]

Supplementary Information

Ultra-thin Solid Electrolyte Interphase Evolution and Wrinkling Processes in Molybdenum Disulfide-based Lithium-ion Batteries

Wan et al.

Supplementary Figures

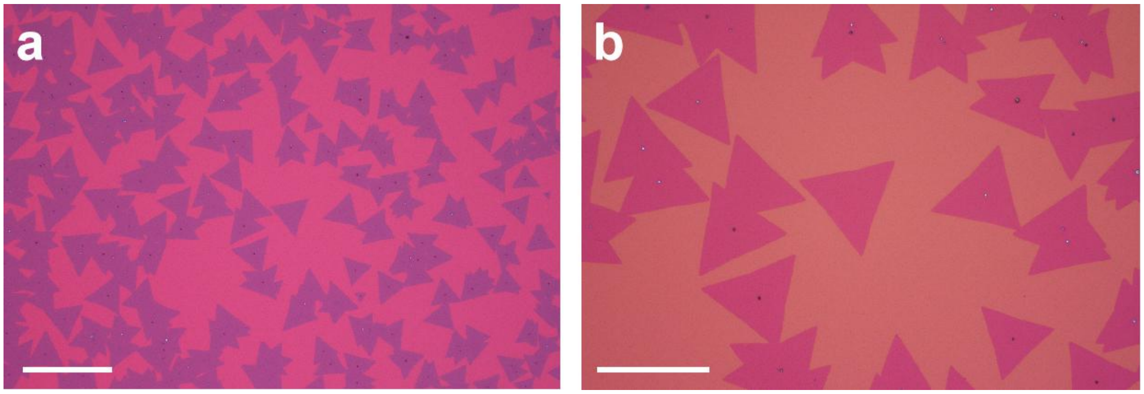

Supplementary Figure 1. The optical images of large-area ultra-flat monolayer MoS_2 on SiO_2 substrate, which is synthesized and fabricated by the chemical vapor deposition (CVD) method. The scale bars are 100 µm in (a) and 50 µm in (b).

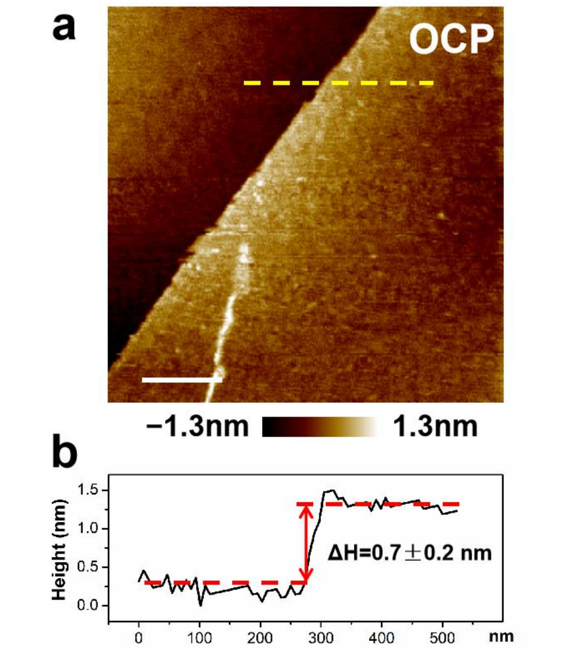

Supplementary Figure 2. The AFM image and height section of the monolayer MoS_2 electrode. (a) AFM image of the MoS_2 electrode produced by CVD and transferred to Si substrate. The scale bar is 400 nm. (b) The thickness of such MoS_2 electrode is measured as 0.7 ± 0.2 nm along the dashed line indicated in (a).

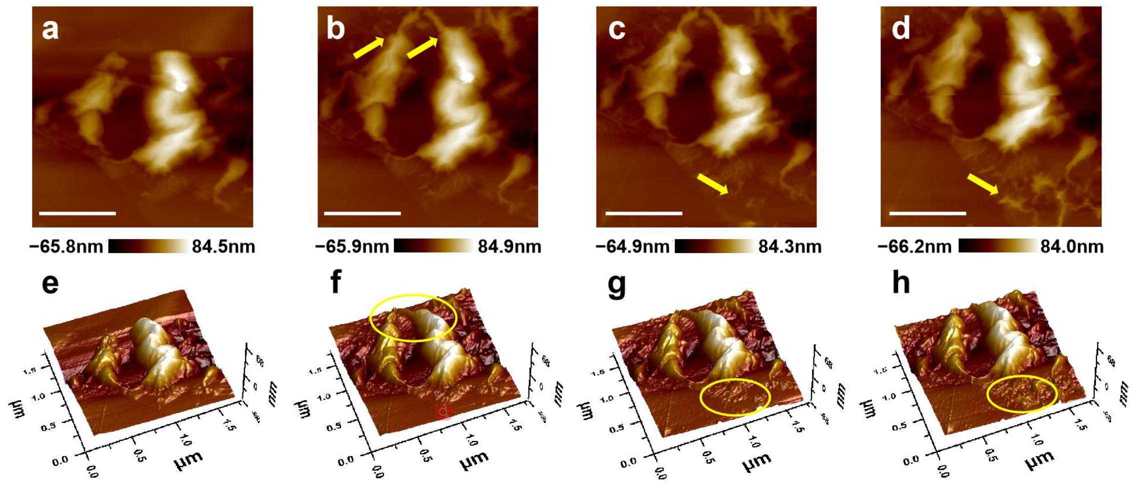

Supplementary Figure 3. *In situ* 2D and corresponding 3D AFM images of the interfacial evolution of wrinkle-structure networks upon lithiation. (a) The initial formation of the planar nanofolds at the early stage of lithiation. Such wrinkling nanostructures grow and propagate at the interface (b, c), and finally appear as the morphology of wrinkle-structure networks (c). Corresponding 3D AFM images (e–h) further manifest the live-formation of network-distributed wrinkles. The apparent variations of nanofolds are indicated by the yellow arrows in b-d and circles in (f–h). The scale bar is 600 nm in (a–d).

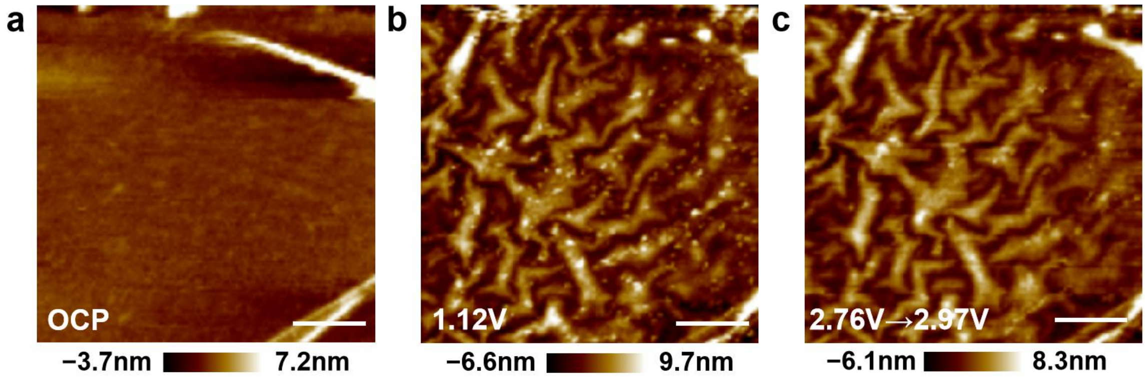

Supplementary Figure 4. *In situ* AFM images of monolayer MoS_2/electrolyte interface in $[BMP]^+[FSI]^-$ containing 0.5 M LiFSI electrolyte in a home-made electrochemical cell. (a) The edge and platform of monolayer MoS_2 are shown at OCP. (b) Lithiation process of MoS_2, which is accompanied by the formation of network wrinkles at cathodic 1.12 V. (c) Wrinkles remain at the interface when discharging from 2.76 V to 2.97 V after delithiation, revealing the capacity fading mechanism of batteries. The scale bar is 300 nm in (a–c).

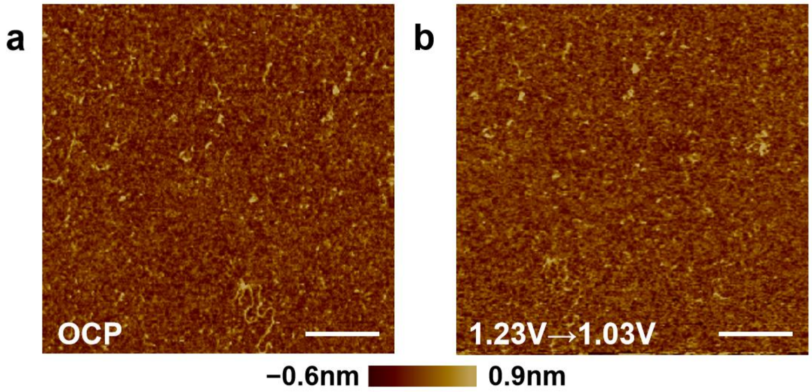

Supplementary Figure 5. *In situ* AFM images of Si (100) electrode in $[BMP]^+[FSI]^-$ containing 0.5 M LiFSI electrolyte (a) at OCP, and (b) charged from 1.23 V to 1.03 V. The scale bar is 200 nm.

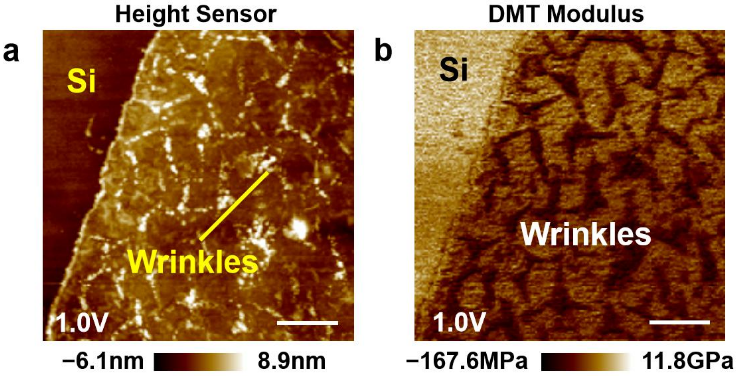

Supplementary Figure 6. Morphology and DMT Modulus of the lithiated monolayer MoS_2 with underlying Si. The AFM images of (a) topography, (b) DMT Modulus and (c) deformation of the wrinkle-like MoS_2 and Si substrate at cathodic 1.0 V. The scale bar is 400 nm.

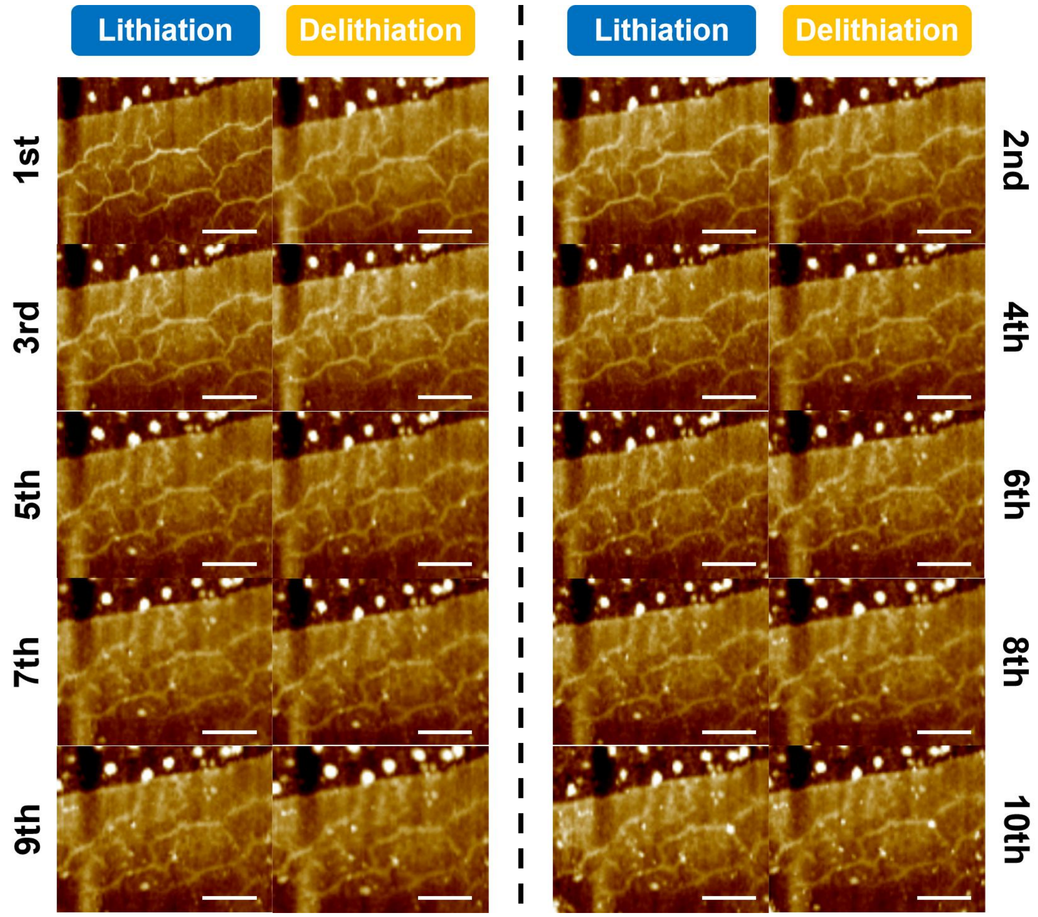

Supplementary Figure 7. *In situ* AFM images of the manolayer MoS_2 electrode/electrolyte interface after lithiation/delithiation in each cycle in the electrolyte with 10 wt% FEC upon 10 cycles. The scale bar is 300 nm.

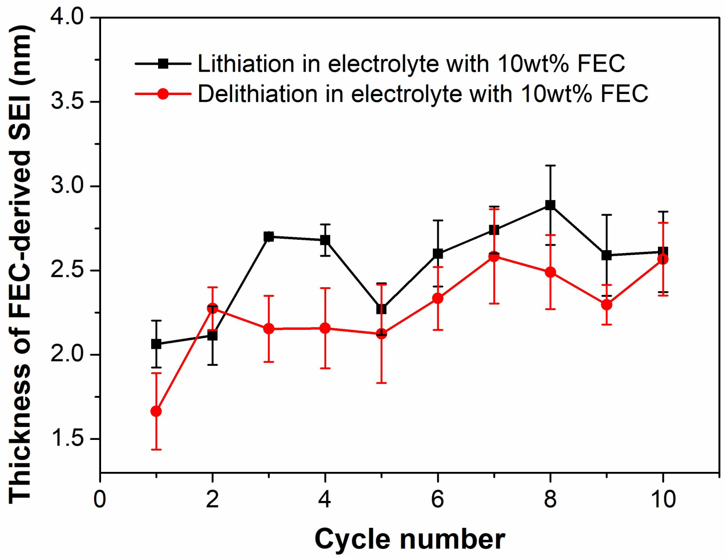

Supplementary Figure 8. Quantitative measurements of the thickness of FEC-derived SEI film after lithiation/delithiation in each cycle upon 10 cycles. And correspondingly specific statistics are shown in Supplementary Table 2.

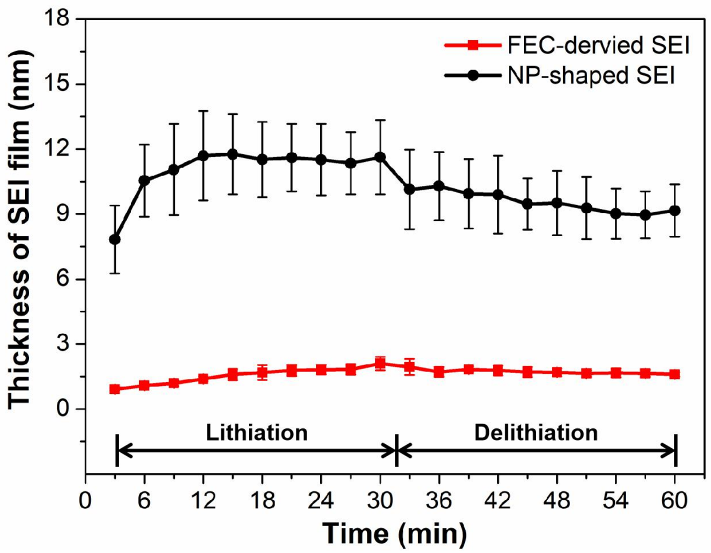

Supplementary Figure 9. Quantitative measurements of the thickness of NP-shaped and FEC-derived SEI films upon lithiation/delithiation processes. Measured 20 successive AFM images are selected in one experiment, in which the first 10 images are captured upon lithiation, and the residual 10 images belong to delithiation process, the Δt of adjacent image is 3 min. Specific statistics are shown in Supplementary Table 3. For NP-shaped SEI film, it exhibits a faster kinetics at the initial growth with the nanoparticle size rapidly growing from 7.8 ± 1.6 nm to 10.5 ± 1.7 nm, and then remains substantially with the size of 11.8 ± 2.1 nm in the subsequent lithiation. Nevertheless, the ultra-thin FEC-derived SEI film almost maintains a thickness of 1.8 ± 0.9 nm upon lithiation, revealing the interphasial homogeneity of the FEC-derived SEI film. During the delithiation process, the thickness of the NP-shaped SEI film instantly decreases from 11.6 ± 1.7 nm to 10.1 ± 1.8 nm, and then remains stable at 9.2 ± 1.2 nm. However, the FEC-derived SEI film substantially maintains with the thickness of 1.7 ± 0.2 nm.

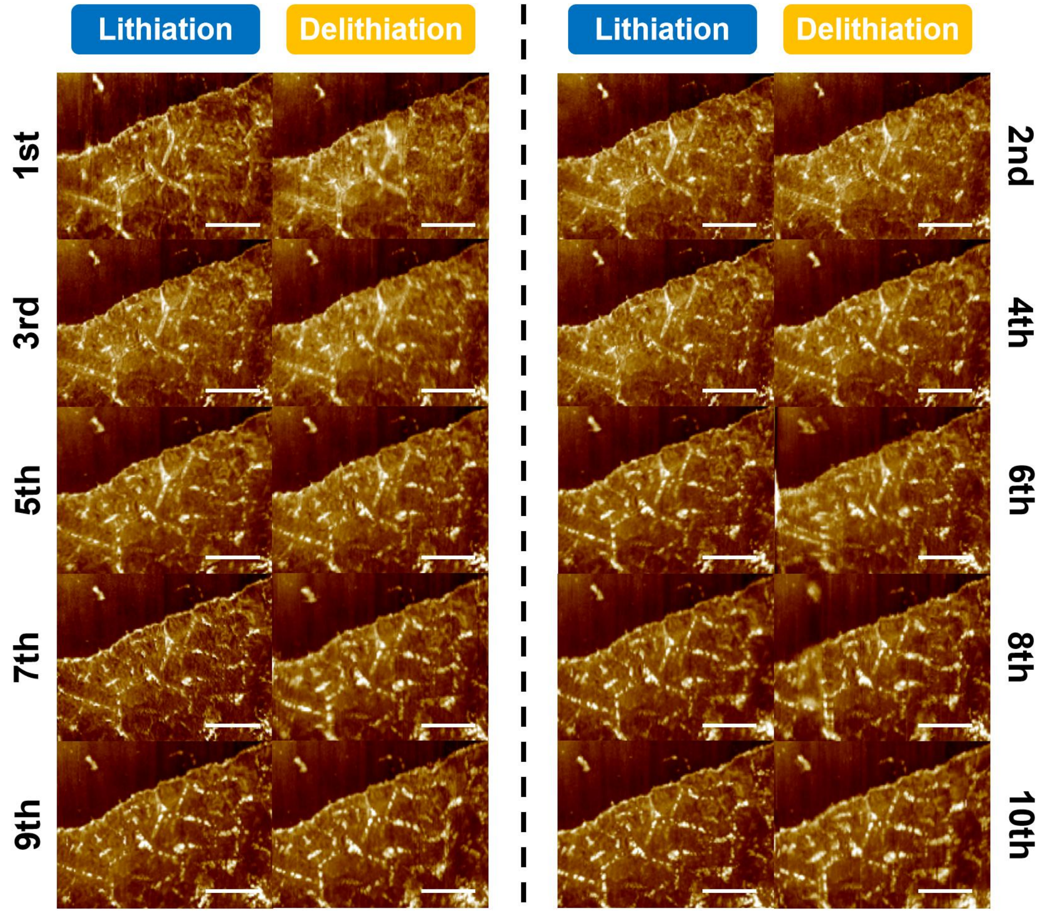

Supplementary Figure 10. *In situ* AFM images of the manolayer MoS_2 electrode/electrolyte interface after lithiation/delithiation in each cycle in the electrolyte without FEC upon 10 cycles. The scale bar is 500 nm.

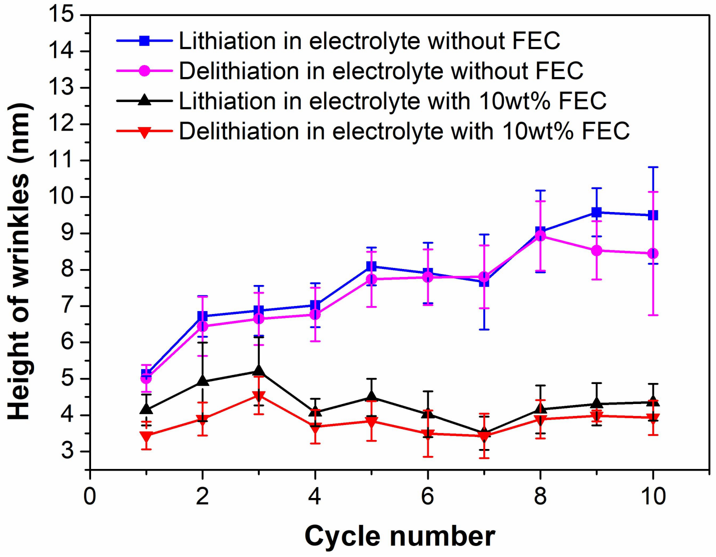

Supplementary Figure 11. Quantitative measurements of the height of wrinkles after lithiation/delithiation in each cycle in the electrolyte with/without 10 wt% FEC upon 10 cycles. And correspondingly specific statistics are shown in Supplementary Table 4. The experimental results indicate that, for FEC-free system, the average height of wrinkles increases from 5.1 ± 0.4 nm of the 1st cycle to 9.0 ± 1.7 nm of the 10th cycle, appearing as a continuously growing and subsequently maintaining trend throughout the cycles. Nevertheless, in FEC-containing system, there is no significant increase in the wrinkle heights with an overall retention of 4.1 ± 0.6 nm, elucidating the surface effect of FEC additive and the cycle stability of electrodes. The larger decay of the wrinkle heights after delithiation in FEC-containing system, further indicating a better reversibility upon cycles.

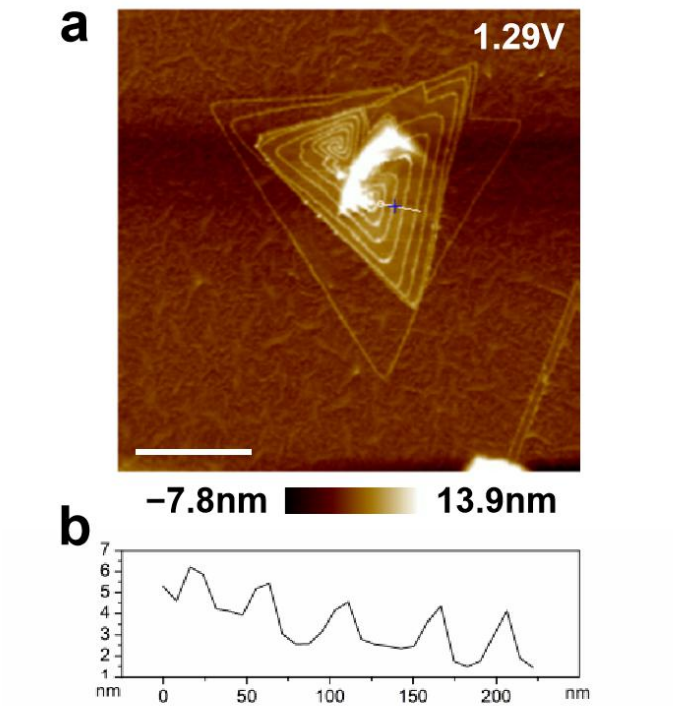

Supplementary Figure 12. The AFM image and corresponding height section of multilayer MoS_2 electrode. The measured position is chosen randomly, and specific height change information (b) is shown beneath the AFM image (a). (a) AFM image of multilayer MoS_2 with edges highlighted at cathodic 1.29 V. (b) The vertical height differences between every two adjacent peaks are 0.7, 0.9 and 0.6 nm, respectively. The scale bar is 500 nm.

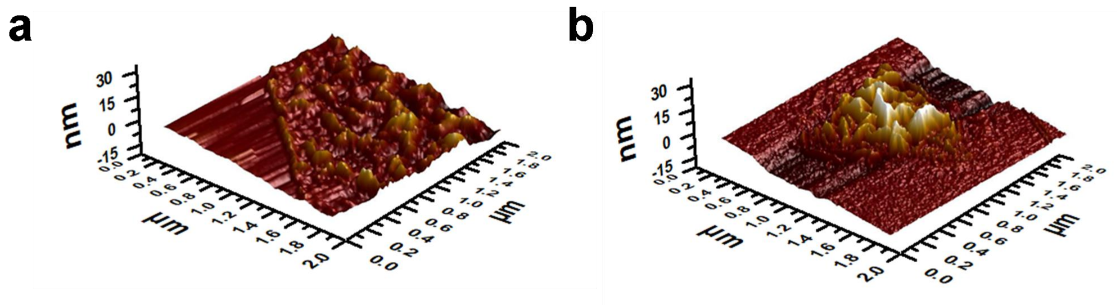

Supplementary Figure 13. Space comparison of wrinkles in monolayer and multilayer MoS_2 systems. 3D AFM images of (a) monolayer and (b) multilayer MoS_2/electrolyte interfaces after lithiation, which are corresponding to images of Figure 1h and 3e in main text, respectively.

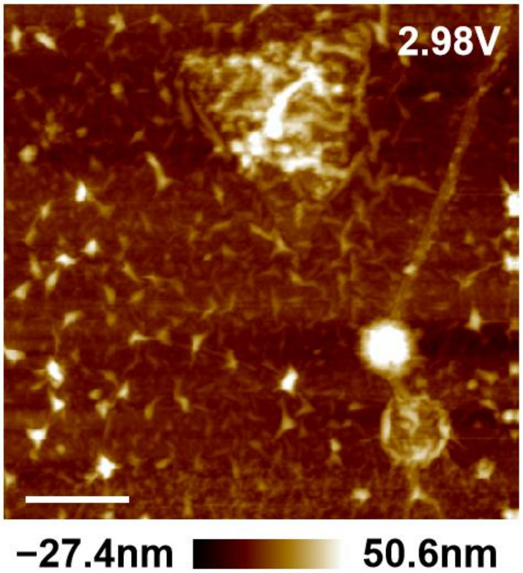

Supplementary Figure 14. *In situ* AFM image of multilayer MoS_2/electrolyte interface in a home-made electrochemical cell in $[BMP]^+[FSI]^-$ containing 0.5 M LiFSI electrolyte at anodic 2.98 V. The scale bar is 600 nm.

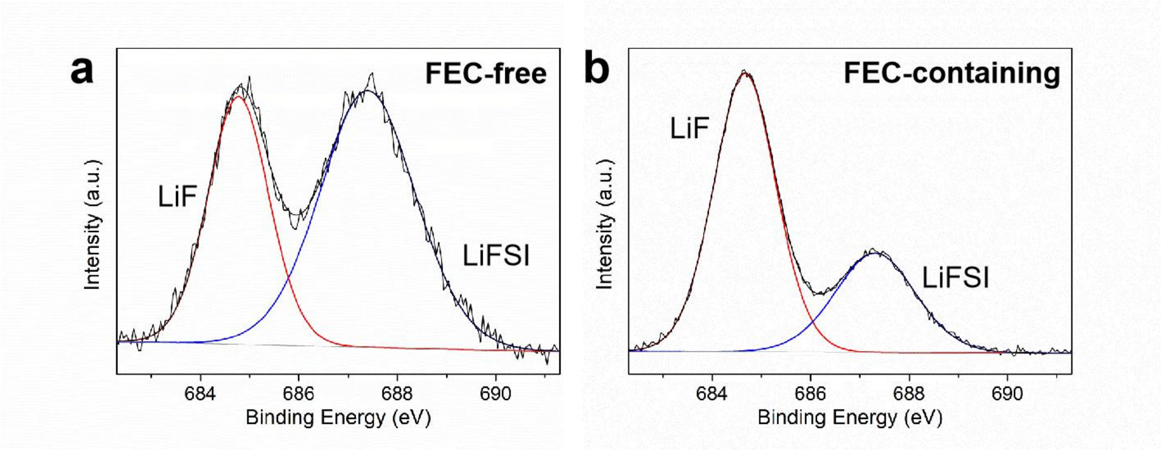

Supplementary Figure 15. Interphasial chemistry of SEI films in the presence and absence of FEC additive. F 1s XPS spectra of samples after lithiation in (a) FEC-free and (b) FEC-containing system. Two peaks at about 684.7 and 687.5 eV are attributed to LiF and LiFSI, and peak area ratios of LiF/LiFSI are 0.62 and 2.18 in (a) and (b), respectively.

Supplementary Tables

Supplementary Table 1. Specific measurement statistics of the thickness of FEC-derived SEI film at a certain position during the whole charging process, where the initial 18 min corresponds to the SEI formation and the left ones belong to the lithiation process.

Time (min)	Thickness of FEC-derived SEI (nm)					Average (nm)	Stdeva (nm)
	1	2	3	4	5		
**3**	0.77	0.39	0.87	0.60	0.48	0.622	0.199
**6**	0.75	0.57	0.88	0.86	0.73	0.758	0.124
**9**	0.66	0.51	0.83	0.74	0.53	0.654	0.136
**12**	0.83	0.65	0.93	0.70	0.64	0.750	0.126
**15**	0.75	0.54	0.87	0.71	0.66	0.706	0.121
**18**	0.73	0.59	0.86	0.73	0.65	0.712	0.102
**21**	0.85	0.66	0.94	0.74	0.68	0.774	0.119
**24**	0.97	0.62	1.18	1.05	0.77	0.918	0.223
**27**	1.15	0.96	1.71	1.27	0.95	1.208	0.311
**30**	1.37	1.14	1.66	1.63	1.24	1.408	0.231
**33**	1.14	0.88	1.45	1.48	1.20	1.230	0.246
**36**	1.45	0.86	1.84	1.41	1.40	1.392	0.349
**39**	1.53	0.75	1.98	1.44	1.63	1.466	0.450
**42**	1.54	0.91	1.99	1.83	1.54	1.562	0.413
**45**	1.83	1.16	2.07	1.73	2.08	1.774	0.375
**48**	1.80	1.14	1.95	2.29	1.82	1.800	0.418
**51**	2.29	1.06	2.91	1.93	1.80	1.998	0.678
**54**	1.88	1.51	2.24	2.13	1.68	1.888	0.304
**57**	1.49	1.54	1.77	2.26	1.78	1.768	0.305
**60**	1.94	1.38	2.58	1.77	2.10	1.954	0.441

Supplementary Table 2. Correspondingly specific statistics of the thickness measurements of the FEC-derived SEI film after lithiation/delithiation of each cycle in 10 cycling processes.

Lithiation					
**Cycle number**	**Thickness of FEC-derived SEI (nm)**			**Average (nm)**	**Stdeva (nm)**
	**1**	**2**	**3**		
**1**	2.29	1.94	1.96	2.06	0.16
**2**	2.37	2.09	1.88	2.11	0.20
**3**	2.72	2.68	2.7	2.70	0.02
**4**	2.78	2.73	2.53	2.68	0.11
**5**	2.52	2.14	2.15	2.27	0.18
**6**	2.92	2.42	2.46	2.60	0.23
**7**	2.96	2.68	2.58	2.74	0.16
**8**	3.17	2.97	2.52	2.89	0.27
**9**	2.94	2.57	2.26	2.59	0.28
**10**	3	2.39	2.44	2.61	0.28

Delithiation					
**Cycle number**	**Thickness of FEC-derived SEI (nm)**			**Average (nm)**	**Stdeva (nm)**
	**1**	**2**	**3**		
**1**	2.02	1.57	1.4	1.66	0.26
**2**	2.48	2.18	2.16	2.27	0.15
**3**	2.46	2.08	1.92	2.15	0.23
**4**	2.54	1.91	2.02	2.16	0.27
**5**	2.56	2.07	1.74	2.12	0.34
**6**	2.63	2.12	2.25	2.33	0.22
**7**	2.97	2.6	2.18	2.58	0.32
**8**	2.74	2.59	2.14	2.49	0.25
**9**	2.43	2.35	2.11	2.30	0.14
**10**	2.9	2.5	2.3	2.57	0.25

Supplementary Table 3. Accordingly specific statistics of the thickness measurements of two distinguishable NP-shaped and FEC-derived SEI films upon lithiation/delithiation, where the initial 30 min corresponds to the lithiation process and the left ones belong to the delithiation process.

Time (min)	Thickness of FEC-derived SEI film (nm)			Average (nm)	Stdeva (nm)	Thickness of NP-shaped SEI film (nm)			Average (nm)	Stdeva (nm)
	1	2	3			1	2	3		
**3**	0.87	0.9	0.98	0.92	0.05	9.11	5.27	9.12	7.83	1.81
**6**	1.02	1.05	1.18	1.08	0.07	10.11	8.43	13.08	10.54	1.92
**9**	1.09	1.11	1.4	1.20	0.14	10.62	8.32	14.22	11.05	2.43
**12**	1.28	1.35	1.56	1.40	0.12	10.68	9.42	14.98	11.69	2.38
**15**	1.25	1.56	1.99	1.60	0.30	10.46	10.06	14.78	11.77	2.14
**18**	1.21	1.66	2.18	1.68	0.40	9.95	10.26	14.34	11.52	2.00
**21**	1.45	1.73	2.18	1.79	0.30	10.14	10.54	14.13	11.60	1.79
**24**	1.46	1.99	2.02	1.82	0.26	9.61	10.8	14.11	11.51	1.90
**27**	1.53	1.76	2.21	1.83	0.28	9.64	10.81	13.59	11.35	1.66
**30**	1.68	2.09	2.54	2.10	0.35	9.6	10.96	14.31	11.62	1.98
**33**	1.47	1.87	2.5	1.95	0.42	7.43	10.39	12.6	10.14	2.12
**36**	1.42	1.67	2.06	1.72	0.26	7.95	10.57	12.37	10.30	1.81
**39**	1.66	1.8	2.06	1.84	0.17	7.45	10.48	11.9	9.94	1.86
**42**	1.46	1.79	2.1	1.78	0.26	7.09	10.61	12	9.90	2.07
**45**	1.36	1.78	2.01	1.72	0.27	7.66	9.76	10.97	9.46	1.37
**48**	1.44	1.69	1.97	1.70	0.22	7.29	9.81	11.45	9.52	1.71
**51**	1.43	1.64	1.87	1.65	0.18	7.03	9.88	10.93	9.28	1.65
**54**	1.38	1.69	1.97	1.68	0.24	7.23	9.4	10.45	9.03	1.34
**57**	1.42	1.65	1.89	1.65	0.19	7.21	9.66	10.03	8.97	1.25
**60**	1.47	1.59	1.75	1.60	0.11	7.34	9.43	10.71	9.16	1.39

Supplementary Table 4. Correspondingly specific statistics of the height measurements of the wrinkles after lithiation/delithiation of each cycle in FEC-free and FEC-containing systems upon 10 cycling tests.

Lithiation

Cycle number	Height of wrinkles in FEC-free system (nm)			Average (nm)	Stdeva (nm)	Height of wrinkles in FEC-containing system (nm)			Average (nm)	Stdeva (nm)
	1	2	3			1	2	3		
**1**	5.19	5.13	5.05	5.12	0.06	4.66	3.48	4.29	4.14	0.49
**2**	7.62	6.14	6.39	6.72	0.65	4.55	3.61	6.59	4.92	1.24
**3**	7.25	5.77	7.59	6.87	0.79	5.23	3.87	6.52	5.21	1.08
**4**	7.73	6.08	7.26	7.02	0.69	4.55	3.5	4.18	4.08	0.43
**5**	8.22	7.3	8.75	8.09	0.60	5.13	3.7	4.63	4.49	0.59
**6**	8.83	6.58	8.32	7.91	0.96	5.03	3.73	3.32	4.03	0.73
**7**	8.55	5.54	8.9	7.66	1.51	4.25	3.06	3.21	3.51	0.53
**8**	10.11	7.23	9.82	9.05	1.29	5.2	3.41	3.86	4.16	0.76
**9**	10.27	8.51	9.96	9.58	0.77	5.22	3.63	4.06	4.30	0.67
**10**	10.29	7.35	10.84	9.49	1.53	4.74	3.53	4.8	4.36	0.59

Delithiation

Cycle number	Height of wrinkles in FEC-free system (nm)			Average (nm)	Stdeva (nm)	Height of wrinkles in FEC-containing system (nm)			Average (nm)	Stdeva (nm)
	1	2	3			1	2	3		
**1**	5.37	5.25	4.41	5.01	0.43	4.05	3.04	3.24	3.44	0.44
**2**	7.71	5.48	6.13	6.44	0.94	4.61	3.69	3.38	3.89	0.52
**3**	7.38	5.49	7.07	6.65	0.83	5.35	3.92	4.36	4.54	0.60
**4**	7.45	5.57	7.28	6.77	0.85	4.34	3.67	3.04	3.68	0.53
**5**	8.2	6.51	8.5	7.74	0.88	4.72	3.3	3.5	3.84	0.63
**6**	7.88	6.67	8.83	7.79	0.88	4.52	3.11	2.85	3.49	0.73
**7**	8.3	6.41	8.7	7.80	1.00	4.22	2.51	3.56	3.43	0.70
**8**	10.15	7.48	9.15	8.93	1.10	4.33	3.03	4.31	3.89	0.61
**9**	9.15	7.22	9.22	8.53	0.93	4.16	3.74	4.04	3.98	0.18
**10**	10.56	5.84	8.94	8.45	1.96	4.08	3.2	4.51	3.93	0.55
