# Supplementary material for: Ultra-thin solid electrolyte interphase evolution and wrinkling processes in molybdenum disulfide-based lithium-ion batteries
Source: Nat Commun. 2019 Jul 22;10:3265. doi: 10.1038/s41467-019-11197-7 (PMC6646323; doi:10.1038/s41467-019-11197-7)
Supplement: Supplementary file 3 — Description of Additional Supplementary Files [file 41467_2019_11197_MOESM3_ESM.pdf]

Description of Additional Supplementary Files

File Name: Supplementary Movie 1

Description: Video of *in situ* AFM recording the initial process of formation of the ultra-thin FEC-derived SEI film upon charging.

File Name: Supplementary Movie 2

Description: Video of *in situ* AFM recording the evolution process of the wrinkle-structure networks upon charging/discharging.
